# Pathological Observations on Fever

**Published:** 1818-10

**Authors:** James Scott


*For the London Medical and Physical Journal.*

### *Pathological Observations on Fever.* By Mr. James Scott.

ALTHOUGH the gleanings of individuals in the medical profession may be unproductive of much general advantage, as solitary cases; yet, when aggregated, and compared with each other, a better illustration may be made of diseases than each case may have afforded by its own lesson: it is by a particular history of each variety and form of a particular disease, that its general and decisive outline is to be obtained; whilst a scientific treatment depends upon a careful observation of the effect of seasons and constitutions in modifying and giving its peculiar character. Every case then, however apparently uninteresting in itself, may acquire an importance, by becoming one of the links in the chain that unites the phenomena which compose the natural history of disease, and connects those miscellaneous observations which, when united, form a faithful history.

Fever, being that departure from health by far the most frequent of any morbid state of the body, attacking indiscriminately all ages, sexes, and conditions, appearing in every country and climate, has claimed the serious attention of those practising the healing art, in an eminent degree; amongst whom, the illustrious Sydenham shines as a star of the first magnitude, and to his discriminating and thinking mind, we are indebted for a more comprehensive view of a disease exhibiting such Proteian qualities. To record, therefore, the progress of so formidable a disease, when assuming a bold and well-defined characteristic, should it add nothing to the general stock of medical knowledge, may possibly keep alive attention to the subject, and elicit observations from those whom experience and talents qualify for the task.

I should not, without these preliminary observations, have brought the following case into notice; for, however desirous I may be of rendering it an exemplification of a material feature in the economy of fever—that of *crisis*, I am aware it may be censured as unimportant. Although the doctrine of concoction has expired, we must still consider the periods denominated crises, to approximate in their *effects* to it, though the *cause* be different; and, although we are not justified in calling in the humoral pathology in explanation of the laws that govern the critical termination of fevers, yet we cannot the less allow such a law obtains, or be less convinced of its sensible effects. Were this circumstance kept in view, more than I think (but I speak with deference,) it is, would it not, I would ask, lead to a corresponding consideration in the treatment? and would not the consequence of this be, that many diseases would be cut short, which would otherwise run a lengthened course? The great Hippocrates was, perhaps, deluded into the error of a fermentation and despumation, by observing the highly morbid state of various evacuations, or the spontaneous discharges, such as hæmorrhage from the nose, flow of urine (possessed of certain qualities), increased perspiration, diarrhœa, &c. which accompanied the crisis, and to which he attributed the crisis itself; assuming that these diseased or spontaneous excretions were the *materies morbi* in a state of concoction, and that their evacuation put an end to that series of actions constituting fever, which he conceived to be merely the efforts of nature to expel some noxious principle. Galen and his followers fell into the same error, and it remained for Cullen to exemplify (what Stahl had previously pointed out,) another principle, which he denominated the "*vis medicatrix naturæ*," and which now stands, in general opinion, almost as highly as its author could have desired. Crisis then may be considered as the acme of the struggle which this vis medicatrix, this preserving power, sets up for the restoration of health, when it either succeeds in restoring the equilibrium upon which health is founded, or having produced a reaction more than commensurate with the powers of the constitution, gives up the contest by a fatal termination. The action of medicines, therefore, on the human constitution, being either to assist the efforts of nature, when judiciously administered, or to embarrass and weaken them, when misdirected, it becomes important that we should regulate our practice, so that it may fall in with the natural efforts of the constitution, observing a careful correspondence between the exhibition of remedies and the periodical actions of the disease. That these actions and periods are much influenced by the operations of medicines is universally known, and some valuable information has lately been afforded on this subject by Dr. Cheyne, of Dublin, in a paper contained in the Dublin Hospital Reports. I cannot forbear quoting one or two passages from this intelligent physician's report:—"The advantage of blood-letting (in fever) appeared not merely in the relief which was immediately experienced by the patient, but in cathartics acting with more ease and effect, in all the surfaces yielding, the skin and mouth becoming moister, *and in a complete and regular crisis taking place very often on the next critical day.*" And again, "In very many instances, the ablution of great part of the body, change of linen, the common purgatives of the hospital, the pure air of the ward, and the supply of cold diluting drinks, *were productive of a crisis in the course of the first night.*" And a little further on, he says, "I believe Sydenham's opinion, that purgatives given at the time of coction prevent crisis, is erroneous, at least in this kind of fever; for purgatives were always given upon admission, and continued generally for twenty-four hours; *and the frequency of crisis in the night after admission was very remarkable.*"

I am compelled abruptly to close these observations, and proceed to the case.

Mr. J——, of the sanguineous temperament, aged thirty-two, after partaking of a moderate supper, and going to-bed in perfect health on the night of the 12th of August last, was attacked, about three o'clock in the morning of the 13th, with a violent rigor, succeeded by intense head-ache and delirium. I did not see him until five o'clock in the afternoon, when I found him with a pulse full, not hard, yet resisting considerably pressure of the finger, beating one hundred and twenty strokes in a minute, the breathing oppressed, and the skin hot and dry; he was then in a state of imperfect delirium, the carotids beating with much violence. He answered coherently to questions put to him, and complained of violent pain in the head; when undisturbed by interrogations, he relapsed into muttering ejaculations, from which, however, he was easily roused by being spoken to. He was blooded to the extent of sixteen ounces, and some purgative pills of calomel and colocynth, assisted with repeated doses of a solution of magnes. sulphat. in infusion of senna, were exhibited; a blister was applied upon the nape of the neck, and his legs plunged into hot water. On the 14th the symptoms continued nearly the same, he had a restless and sleepless night, delirium continued, head-ache intense, pulse somewhat accelerated. As the purgative had acted but imperfectly, it was repeated. The blister had produced no vesication, it was therefore removed, and replaced by another, whose stimulating effects were increased by covering its surface with finely powdered cantharides. A draught of liq. ammon. acet. mistur. camphor, with tinct. digital. gtt. xx. was given every five hours. Fourteen ounces of blood were again taken from the arm.—On the 15th he had every symptom remaining in its full force, yet the bowels had been opened freely. The blister had not acted, and was therefore continued; he was again blooded, and the digitalis persisted in; feet again immersed in hot water. He was somewhat better on the 16th, had gotten some sleep, and remained more free from delirium, although he still possessed that aberration of mind, which kept him wandering and uttering unmeaning exclamations; his head-ache was still very severe, the pulse had sunk to one hundred and ten, the skin had become moist and perspirable, the bowels had been opened but once; the tongue remained coated with a thick buffy coat, and the fœtor of the breath was excessive. The purgative medicines were again administered, and the digitalis draughts continued. 17th, bowels had been acted on by the purgative, which had produced several dark fœtid motions; the pulse had sunk to one hundred, and beat with less violence, and had lost a jirk which it hitherto had. The skin was moist, and he had sweated profusely for several hours during the night, he complained of much debility, which he attributed to his abstinence from food: he was ordered a mixture of mistur. camphoræ, infus. sennæ p. æ. in which was dissolved a dram of the nitrate of potash, to be taken at intervals; some broth and jelly were allowed him.

At three o'clock in the morning of the 18th, precisely the same hour of the attack, he was seized with a violent rigor, attended with increased delirium, which lasted two hours; the skin gradually relaxed, and a gentle moisture was followed by a profuse sweat; the head-ache diminished, and gradually disappeared; he became calm, and, under a state of pleasurable sensation, he sunk into a profound sleep, from which, in the morning, he awoke convalescent, the disease having ran a period of five days. He continued gradually to recover his health and strength, under a tolerably liberal diet and the use of the bark, until the morning of the 23d, which was another period of five days from the day of the crisis; when, at the usual hour of three o'clock, he was attacked with rigor, and went through every successive stage in the same manner and with the same violence as in the first attack. The history of the following days would be but a repetition of the first interval: the disease shewed precisely the same phenomena, and the treatment adopted was similar to the preceding, regulated only according as the time and circumstances might a little vary with each other. Having seen the law which had so clearly obtained in this disease of an observance of a period of five days, both in the crisis and in the relapse, I could not forbear expecting that the next critical period on the fifth day would be attended also with as marked an exacerbation, and I ventured to hint to the friends of the patient, on the day of the 27th, that the next morning might probably produce a decisive effect; and this expectation was happily accomplished under the same violent impression, and with the same concomitant circumstances, which marked the former crisis.

I have been imperceptibly led so far, that I cannot now offer the remarks on this case which I had at first intended, but shall leave it, without farther comment, for your readers' attention.

*London; Sept.* 9, 1818.

